# Comparative effects of different posterior decompression techniques for lumbar spinal stenosis: a systematic review and Bayesian network meta-analysis

**DOI:** 10.1186/s13018-024-04792-y

**Published:** 2024-07-20

**Authors:** Kun Wu, Zhihe Yun, Jun Zhang, Tao Yu, Anyuan Dai, Yang Sun, Chen Li, Yanli Wang, Qinyi Liu

**Affiliations:** Department of Orthopaedics, The Second Hospital of Jilin University, Changchun, Jilin Province China

## Abstract

**Study design:**

A systematic review and Bayesian network meta-analysis (NMA).

**Objective:**

To compare the effectiveness and safety of different posterior decompression techniques for LSS.

**Summary of background data:**

Lumbar spinal stenosis (LSS) is one of the most common degenerative spinal diseases that result in claudication, back and leg pain, and disability. Currently, posterior decompression techniques are widely used as an effective treatment for LSS.

**Methods:**

An electronic literature search was performed using the EMBASE, Web of Science, PubMed, and Cochrane Library databases. Two authors independently performed data extraction and quality assessment. A Bayesian random effects model was constructed to incorporate the estimates of direct and indirect treatment comparisons and rank the interventions in order.

**Results:**

In all, 14 eligible studies comprising 1,260 patients with LSS were included. Five interventions were identified, namely, spinal processes osteotomy (SPO), conventional laminotomy/laminectomy (CL), unilateral laminotomy/laminectomy (UL), bilateral laminotomy/ laminectomy (BL), and spinous process-splitting laminotomy/laminectomy (SPSL). Among these, SPO was the most promising surgical option for decreasing back and leg pain and for lowering the Oswestry Disability Index (ODI). SSPL had the shortest operation time, while SPSL was associated with maximum blood loss. SPO and UL were superior to other posterior decompression techniques concerning lesser blood loss and shorter length of hospital stay, respectively. Patients who underwent BL had the lowest postoperative complication rates.

**Conclusion:**

Overall, SPO was found to be a good surgical choice for patients with LSS.

**Supplementary Information:**

The online version contains supplementary material available at 10.1186/s13018-024-04792-y.

## Introduction

With a rise in the proportion of older individuals in the population, lumbar spinal stenosis (LSS) has gradually emerged as one of the most common degenerative spinal diseases. Pain in the back and leg, claudication, and even disability may occur in these patients [1–5]. In the United States, > 30,000 surgeries for LSS were performed and the gross hospital spending for LSS operations in Medicare alone reached nearly $1.65 billion in 2007 [6]. Thus, LSS imposes a considerable burden on patients and society.

Generally, LSS is treated surgically using posterior decompression techniques and conventional laminectomy has always remained the reference standard [7–10]. However, newer posterior decompression techniques can greatly minimize tissue damage and provide more spinal stability [11, 12]. Currently, the main techniques of posterior decompression can be divided into five categories: conventional laminotomy/laminectomy (CL), unilateral laminotomy/laminectomy (UL), bilateral laminotomy/laminectomy (BL), spinous process splitting laminotomy/laminectomy (SPSL), and spinous process osteotomy (SPO). However, the results of effectiveness and safety evaluations of these posterior decompression techniques for LSS are inconsistent, which necessitates the requirement for evidence-based clinical practice guidelines. There is no comprehensive study comparing all posterior decompression techniques to determine which technique is most beneficial to patients with LSS. Although there are several meta-analyses on the topic, they are all pairwise comparisons of the posterior decompression techniques [7, 13–15]. Therefore, a network meta-analysis (NMA) is necessary. NMA is an expansion of traditional pairwise meta-analyses that can extract and compare clinical trial data, and further incorporate both direct and indirect information to deduce the effectiveness of interventions [16, 17]. Therefore, this study performed a comprehensive NMA to compare the effectiveness and safety of different posterior decompression techniques.

## Methods

### Search strategy

This systematic review and NMA were performed in accordance with the PRISMA guidelines [18–20]. The methods of this review were prospectively registered with PROSPERO (number CRD42022369923). We performed a comprehensive electronic search of PubMed, Embase, Cochrane Library, and Web of Science databases from inception until October 2022. The search strategy is described in Supplementary Data. We also hand-checked the references from the published pairwise meta-analyses to gain relevant articles.

### Selection criteria and research design

The inclusion criteria were as follows: randomized controlled trials (RCTs) of patients with LSS and the included article had to compare at least two posterior decompression techniques for LSS, including CL, UL, BL, SPSL, and SPO. The exclusion criteria were as follows: a follow-up period of < 12 months and patients undergoing reoperation or secondary surgery.

The primary outcomes were as follows: (1) Pain intensity, as measured using the visual analog scale (VAS) [21]. (2) Disability, as measured using the Oswestry Disability Index (ODI) [22–24].

The secondary outcomes were as follows: (1) Perioperative blood loss, (2) operation time, (3) length of hospital stay, and (4) complications.

### Data extraction and assessment for the risk of bias

Data collection was completed independently by two investigators (KW and ZHY). Details of the author, year of publication, study design, diseases, interventions, number of patients, age and gender of patients, and the time of follow-up were collected. Any disagreements were resolved by discussion with the third investigator (QYL). All the included RCTs were assessed for risk of bias using the Cochrane Risk of Bias Tool [25].

### Statistical analysis

We used Stata version 17.0 to conduct a pairwise meta-analysis [26]. NMA was performed using R version 4.2.1 using gemtc and BUGSnet packages. Statistical analyses were performed using Review Manager software (version 5.3) [27]. The data were summarized using the odds ratio (OR) for categorical variables and mean differences (MDs) for continuous data [28, 29].

## Results

### Systematic review

**Fig. 1:**
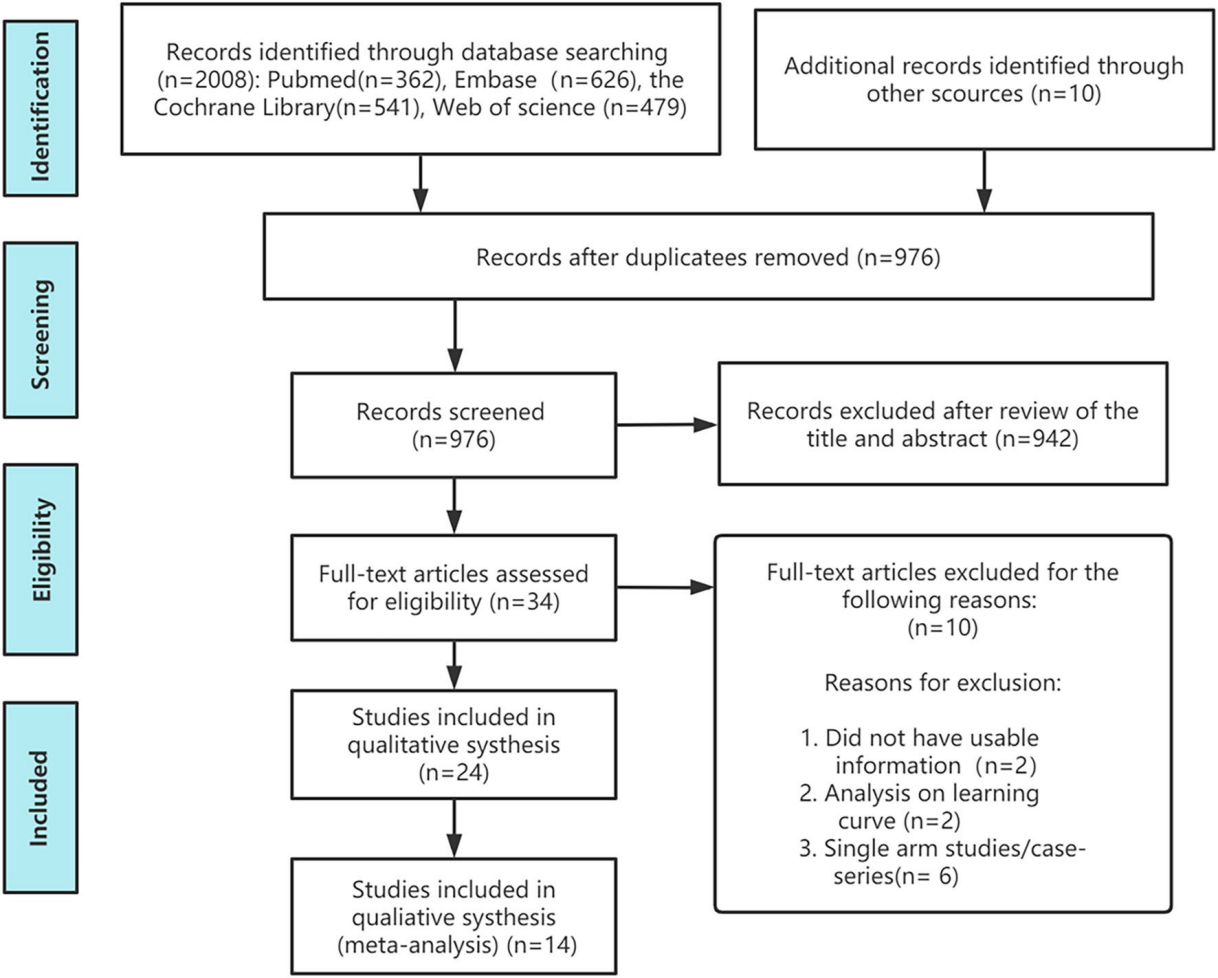
Flow chart of the selection process for relative studies in meta-analysis

**Fig. 2:**
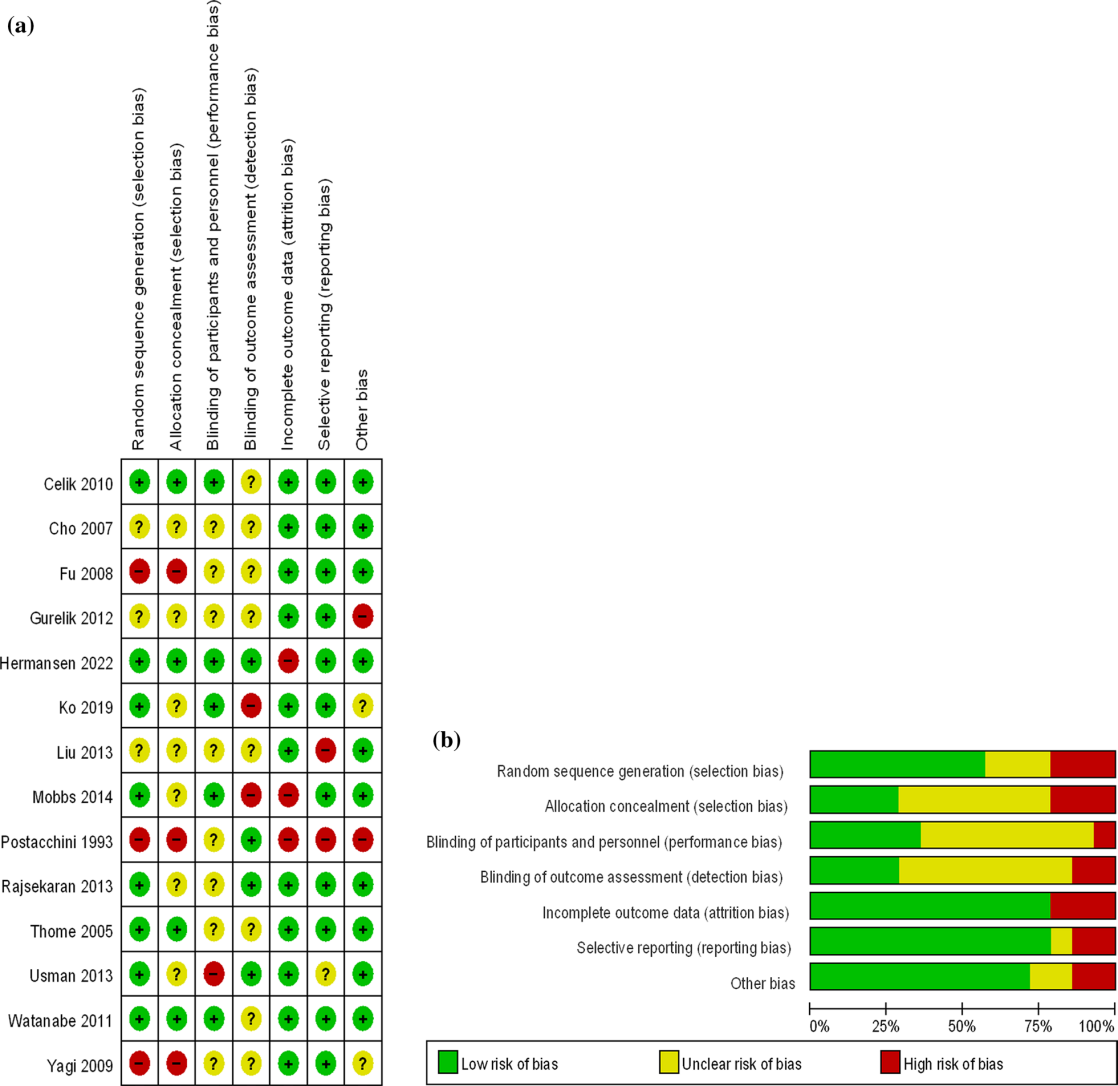
Risk of bias summary for RCTs: Reviewers' judgments about each risk of bias item per included study.

**Fig. 3:**
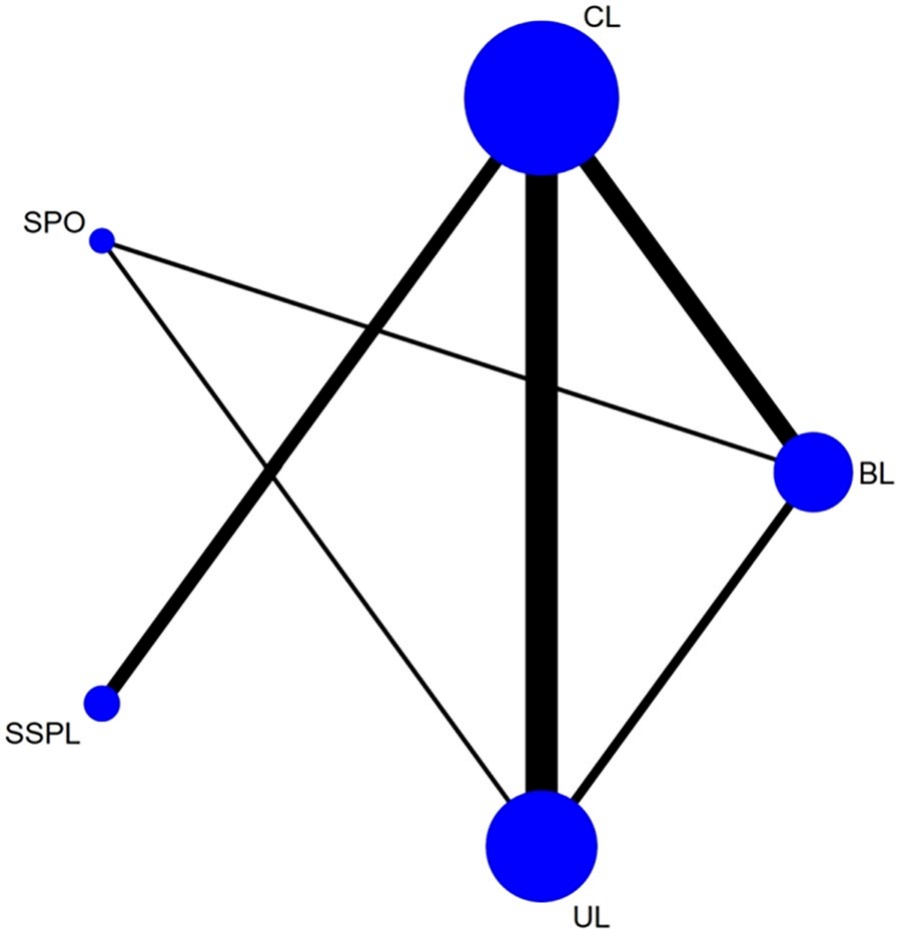
The network plot of all trials

The flowchart for the selection procedure of RCTs is shown in Fig. 1. In all, 14 RCTs (n = 1,260) on five posterior decompression techniques (CL, UL, BL, SPSL, and SPO) were included [30–43]. Table 1 shows the 14 RCTs evaluating five posterior decompression techniques that were included in the NMA. Figure 2 depict the risk of bias assessment for all the included RCTs. The follow‐up period for the primary outcome (Back VAS, Leg VAS, and ODI) was 12 months (The number of literatures with follow-up time over 24 months is relatively small compared with 12 months. For the reliability of the final results, we use the results of the 12th month as a discussion). Figure 3 shows the network plot of all trials. Supplementary Table 1 compares the deviance information criteria (DIC) between the consistency and inconsistency models (greater similarity of the DIC value in the consistent model compared with that in the inconsistent model indicated better consistency) [44, 45]. Supplementary Fig. 1 shows the results of forest map for all outcomes. The node-splitting method showed no significant inconsistency (P > 0.05) and statistical results showed that the inconsistency model accorded well with the consistency model for all outcomes. Supplementary Fig. 2 shows the network plot of all outcomes. The size of the nodes relates to the number of participants in that surgical procedure type and the thickness of lines between surgical procedures relates to the number of studies for that comparison. Supplementary Fig. 3 shows the funnel plot of all outcomes in order to do bias analysis. Publication bias was examined through visual inspection of funnel plot asymmetry. Eventually we found that there was no publication bias in the inclusion of studies in different outcomes.

**Table 1:** The main features of the articles included in the network meta-analysis

Study	Design study (LOE)	Disease	treatment vs comparator	Sample size	gender ( male/female)	Age(Mean ± SD)	follw-up
Celik 2010 [30]	RCT(I)	lumbar spinal stenosis	BL/CL	71	33/38	59.96 ± 13.47	12, 24
Cho 2007 [31]	RCT(I)	lumbar spinal stenosis	SSPL/CL	70	31/39	60.14 ± 12.8	12, 24
Fu 2008 [32]	RCT(I)	lumbar spinal stenosis	BL/CL	152	70/82	57.63 ± 4.98	6, 12, 24
Gurelik 2012 [33]	RCT(I)	lumbar spinal stenosis	UL/CL	52	21/31	64.5 ± 10.06	12, 24
Liu 2013 [36]	RCT(I)	lumbar spinal stenosis	UL/CL	56	30/26	60.04 ± 4.04	12,24
Postacchini 1993 [38]	RCT(II)	lumbar spinal stenosis	BL/CL	70	34/36	57 ± 9	6, 12, 24
Rajsekaran 2013 [39]	RCT(I)	lumbar spinal stenosis	SSPL/CL	51	26/25	56.04 ± 9.97	12, 24
Thome 2005 [40]	RCT(I)	lumbar spinal stenosis	UL/BL/CL	110	53/67	68.68 ± 8.64	12
Watanabe 2011 [42]	RCT(I)	lumbar spinal stenosis	SSPL/CL	34	18/16	69.94 ± 9.04	12
Yagi 2009 [43]	RCT(I)	lumbar spinal stenosis	UL/CL	41	14/27	NR	3,6,12
Ko 2019 [35]	RCT(I)	lumbar spinal stenosis	UL/CL	50	18/32	67.16 ± 9.45	6,12,24
Mobbs 2014 [37]	RCT(I)	lumbar spinal stenosis	UL/CL	54	12/36	69.25 ± 12.86	6,12,24
Usman 2013 [41]	RCT(I)	lumbar spinal stenosis	UL/CL	60	18/12	NR	> 3
Hermansen 2022 [34]	RCT(I)	lumbar spinal stenosis	SPO/UL/BL	437	206/230	68 ± 3.04	12, 24

^*^LOE = Level of Evidence, CL = laminotomy/laminectomy, UL = unilateral laminotomy/laminectomy, BL = bilateral laminotomy/laminectomy, SPSL = spinous process splitting laminotomy/laminectomy, SPO = spinous process osteotomy

### Change in pain scores

#### VAS of the back pain

**Fig. 4:**
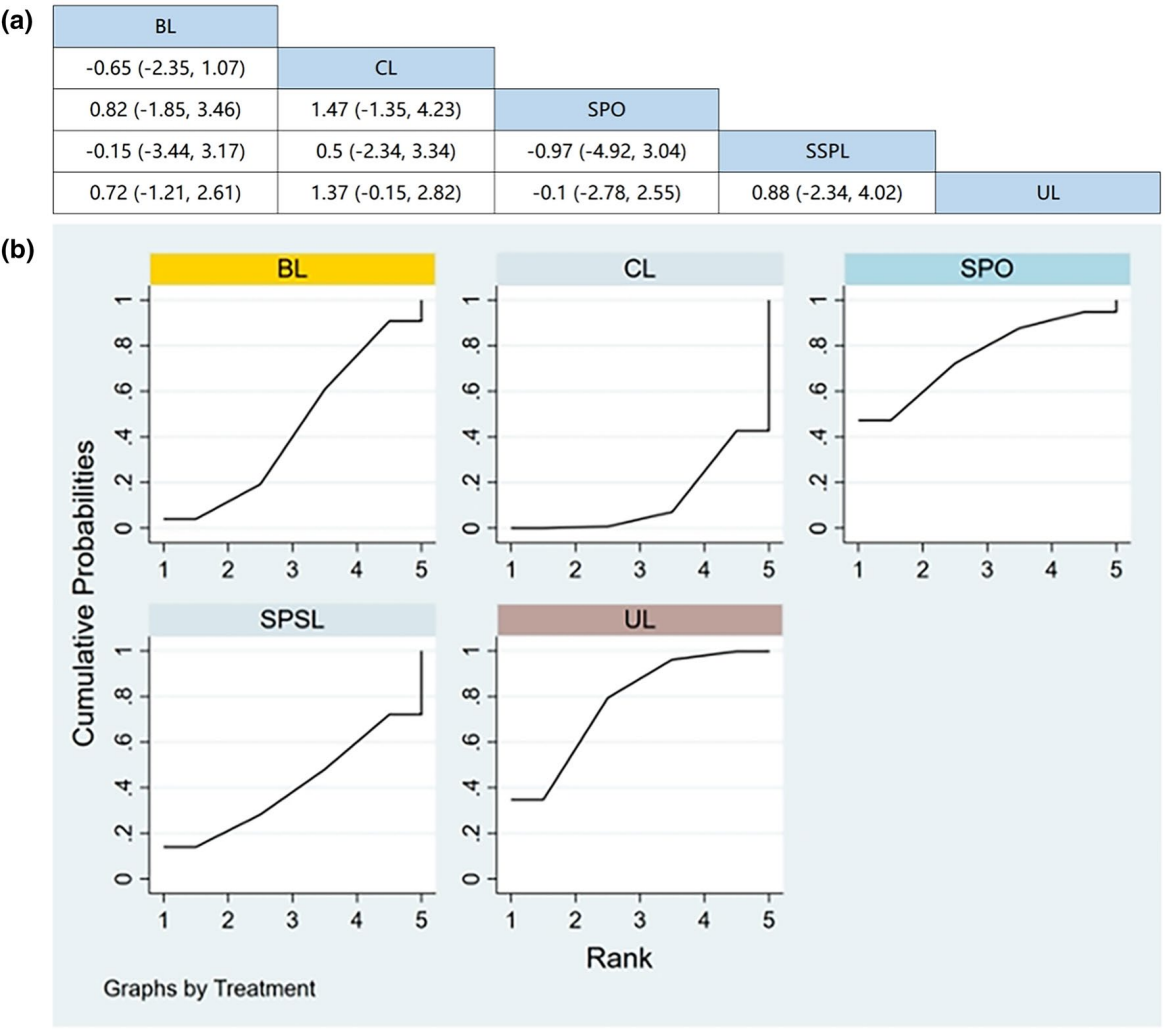
**a** The results of League table for VAS of the back pain. **b** Ranking the probability of back pain change

Seven RCTs (including data from 847 participants) compared the change in back pain among different posterior decompression techniques [30, 32, 34–36, 39, 43]. SPO was found to be superior to other surgeries in relieving back pain. However, Fig. 4a demonstrates that other than SPO, no significant differences were found in pain relief between any two posterior decompression techniques.

#### Ranking the probability of back pain change

We ranked different posterior decompression techniques using the surface under the cumulative ranking curves (SUCRA). Figure 4b shows the outcome of back pain relief (red stands for the first rank, blue for second, and yellow for third). A higher ranking represents the greater effectiveness of the posterior decompression technique. The probability of change in back pain after various posterior decompression techniques ranked from high to low was as follows: BL (59.4%), SPO (59.1%), CL (52.7%), SPSL (40.2%), and UL (38.6%).

#### VAS for leg pain

**Fig. 5:**
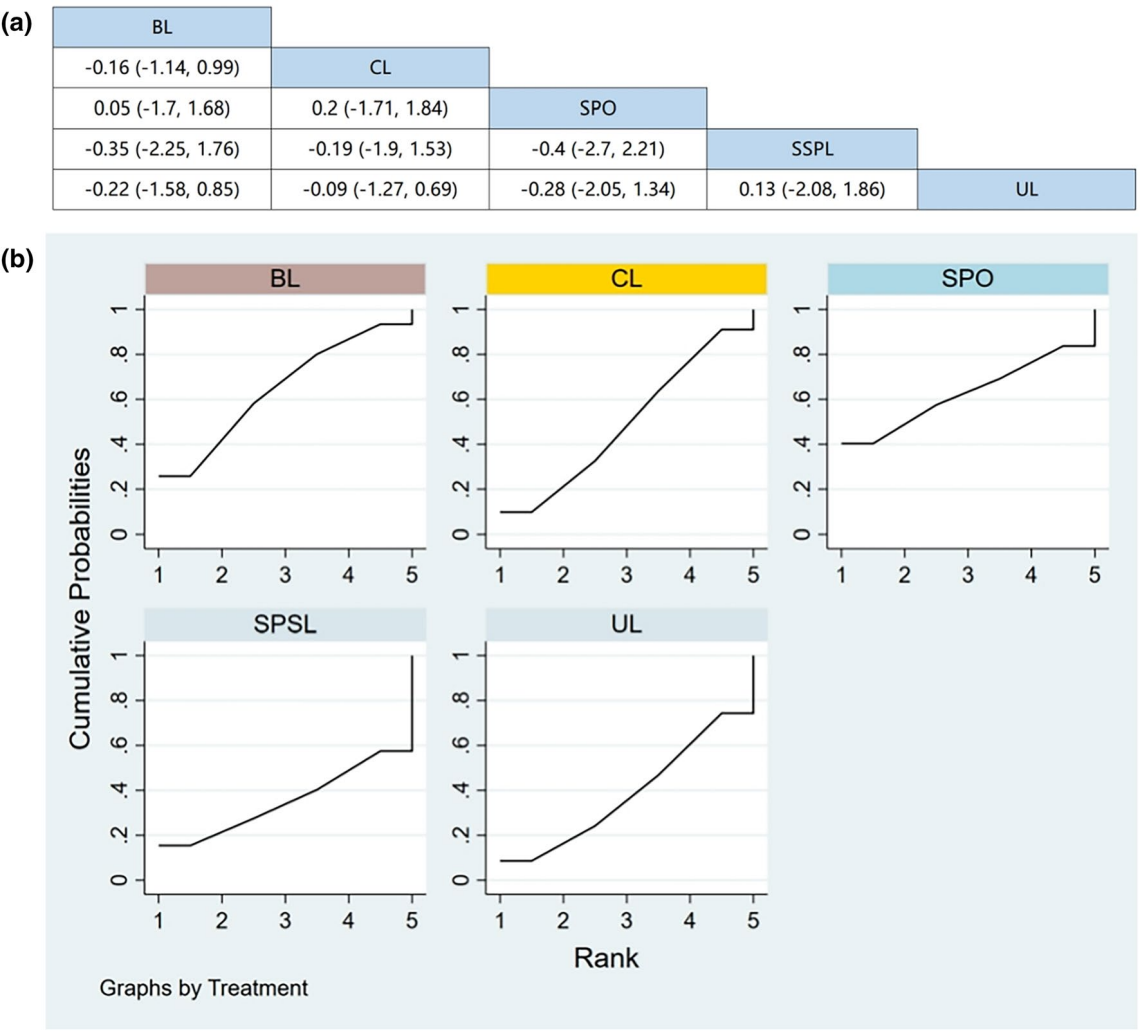
**a** The results of League table for VAS for leg pain. **b** Ranking the probability of leg pain change

Seven RCTs (including data from 835 participants) compared the change in leg pain among different posterior decompression techniques [30, 32, 34–37, 39]. SPO performed better than other surgeries in relieving leg pain. However, Fig. 5a demonstrates that apart from SPO, the mean difference in leg pain relief was not significant between any two surgical interventions.

#### Ranking the probability of leg pain change

We ranked different posterior decompression techniques using SUCRA. Figure 5b shows the outcome of leg pain relief. The probability of change in leg pain after various posterior decompression techniques ranked from high to low was as follows: BL (59.4%), SPO (59.1%), CL (52.7%), SPSL (40.2%), and UL (38.6%).

### Disability change

#### ODI

**Fig. 6:**
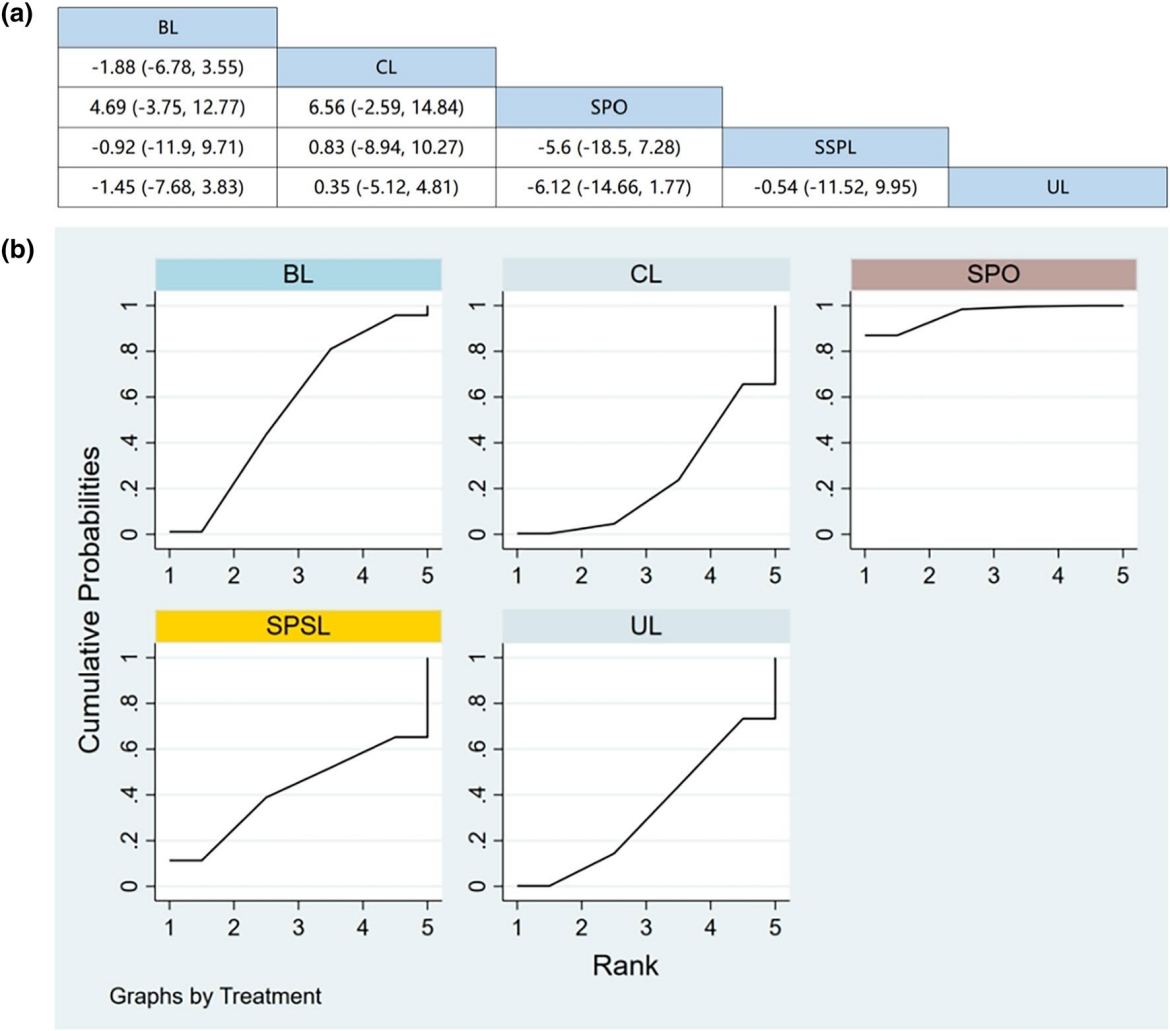
**a** The results of League table for ODI. **b** Ranking the probability of ODI change

Eleven RCTs (including data from 1,361 participants) compared the change in ODI among different posterior decompression techniques [30, 32–37, 39, 40, 42, 43]. SPO had the best effect in reducing ODI. However, Fig. 6a demonstrates that apart from SPO, there were no significant differences in the ODI between any two surgical interventions in the consistency model.

#### Ranking the probability of ODI change

We ranked different posterior decompression techniques using SUCRA. Figure 6b shows the outcome of ODI change. The probability of disability change after various posterior decompression techniques ranked from high to low were as follows: SPO (86.6), BL (53.1%), SPSL (44.9%), UL (32.9%), and CL (32.5%).

#### Blood loss and operation time

**Fig. 7:**
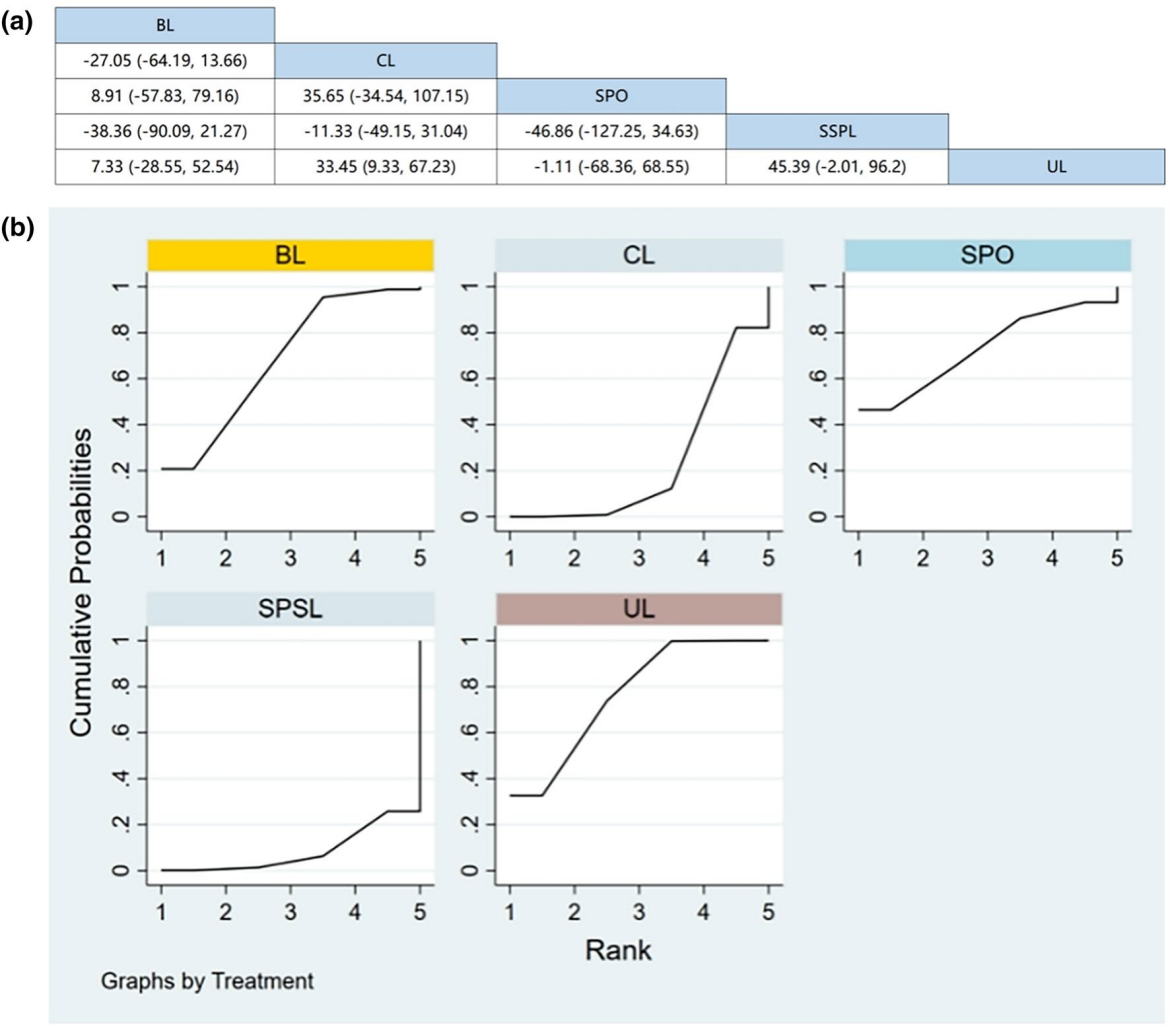
**a** The results of League table for blood loss. **b** Ranking the probability of blood loss

**Fig. 8:**
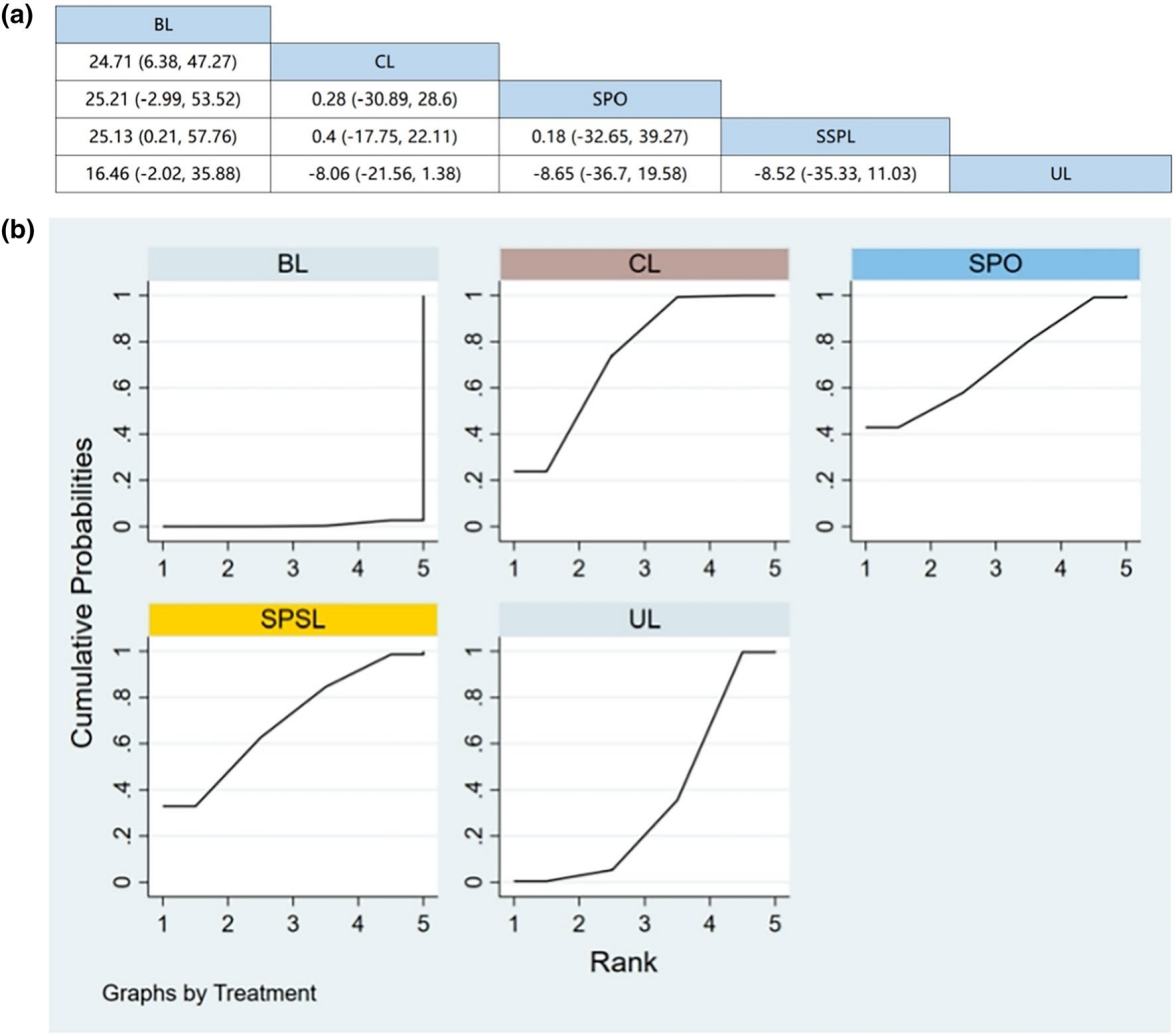
**a** The results of League table for operation time. **b** Ranking the probability of operation time

Seven RCTs (including data from 764 participants) compared blood loss [30, 34, 36, 39, 40, 42, 43] and eight RCTs (including data from 803 participants) compared operation time [34–36, 39–43] among different posterior decompression techniques. Blood loss was higher with SSPL and CL than that with any other surgery. However, SSPL took lesser time to complete than any other surgery. Figures 7a and 8a demonstrate that other than these, there were no significant differences in blood loss and operation time between any two different interventions.

#### Ranking the probability of blood loss and operation time

We ranked different posterior decompression techniques using SUCRA. Figures 7b and 8b show the outcome of blood loss and operation time, with a higher ranking representing greater safety. The probability of blood loss after posterior decompression techniques when ranked from low to high were as follows: UL (81.9%), SPO (59.6%), BL (56.7%), CL (37.1%), and SPSL (14.7%). In addition, the probability of the operation time when ranked from low to high was as follows: CL (74.3%), SPO (70.1%), SPSL (69.1%), UL (35.2%), and BL (0.8%).

#### Hospitalization time

**Fig. 9:**
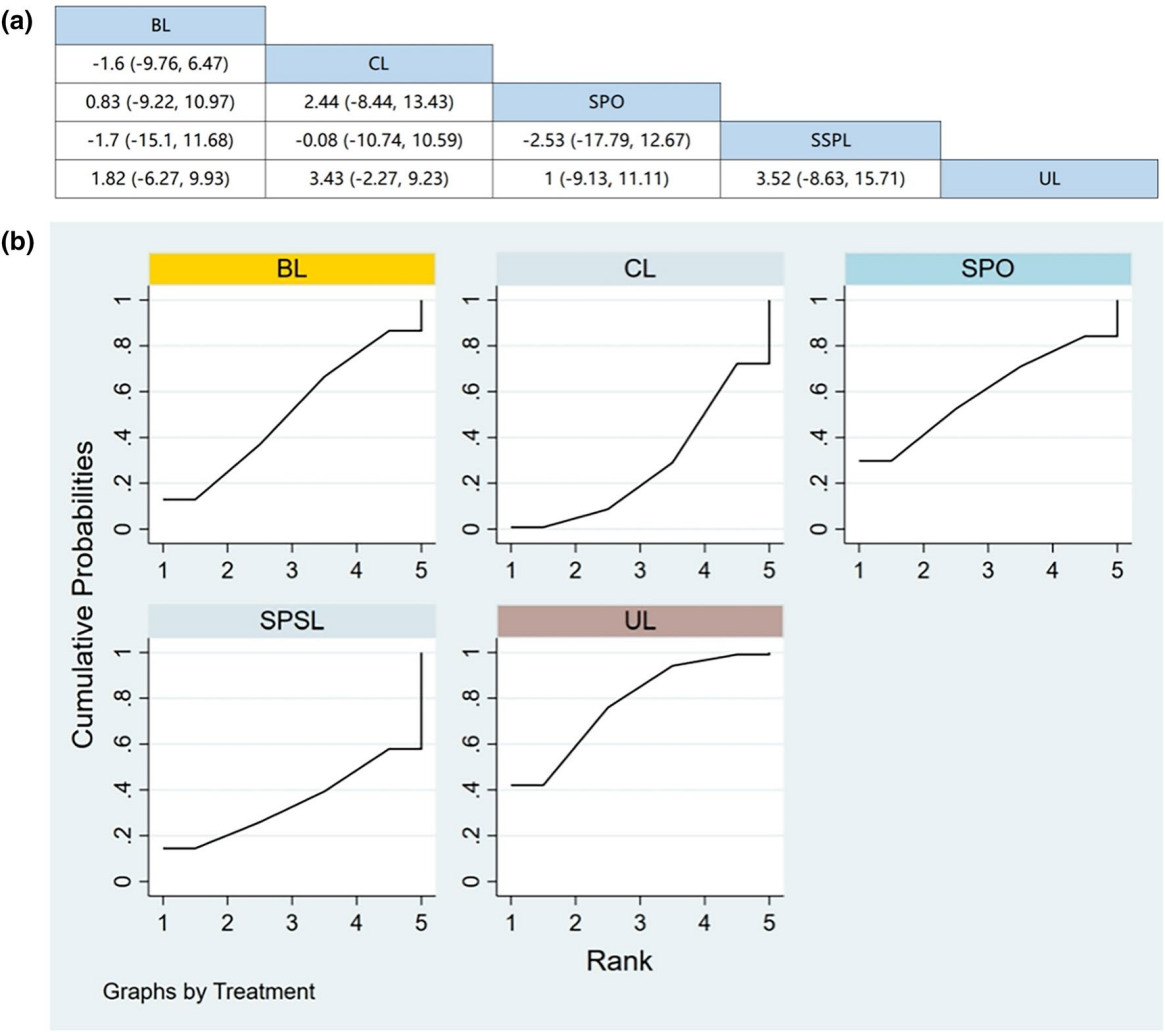
**a** The results of League table for hospitalization time. **b** Ranking the probability of hospitalization time

Six RCTs (including data from 678 participants) compared hospitalization time among different posterior decompression techniques [30, 34, 37, 39, 41, 43]. UL had a shorter duration of hospitalization compared with other surgical interventions. However, Fig. 9a demonstrates that other than UL, there were no significant differences in the hospitalization time between any two interventions.

#### Ranking the probability of hospitalization time

We ranked different posterior decompression techniques using SUCRA. Figure 9b shows the outcome of hospitalization time, with a higher ranking representing more safety. The probability of the hospitalization time ranked from low to high was as follows: UL (75.9%), SPO (66.7%), BL (38.8%), SPSL (36.3%), and CL (32.3%).

#### Complication rate

**Fig. 10:**
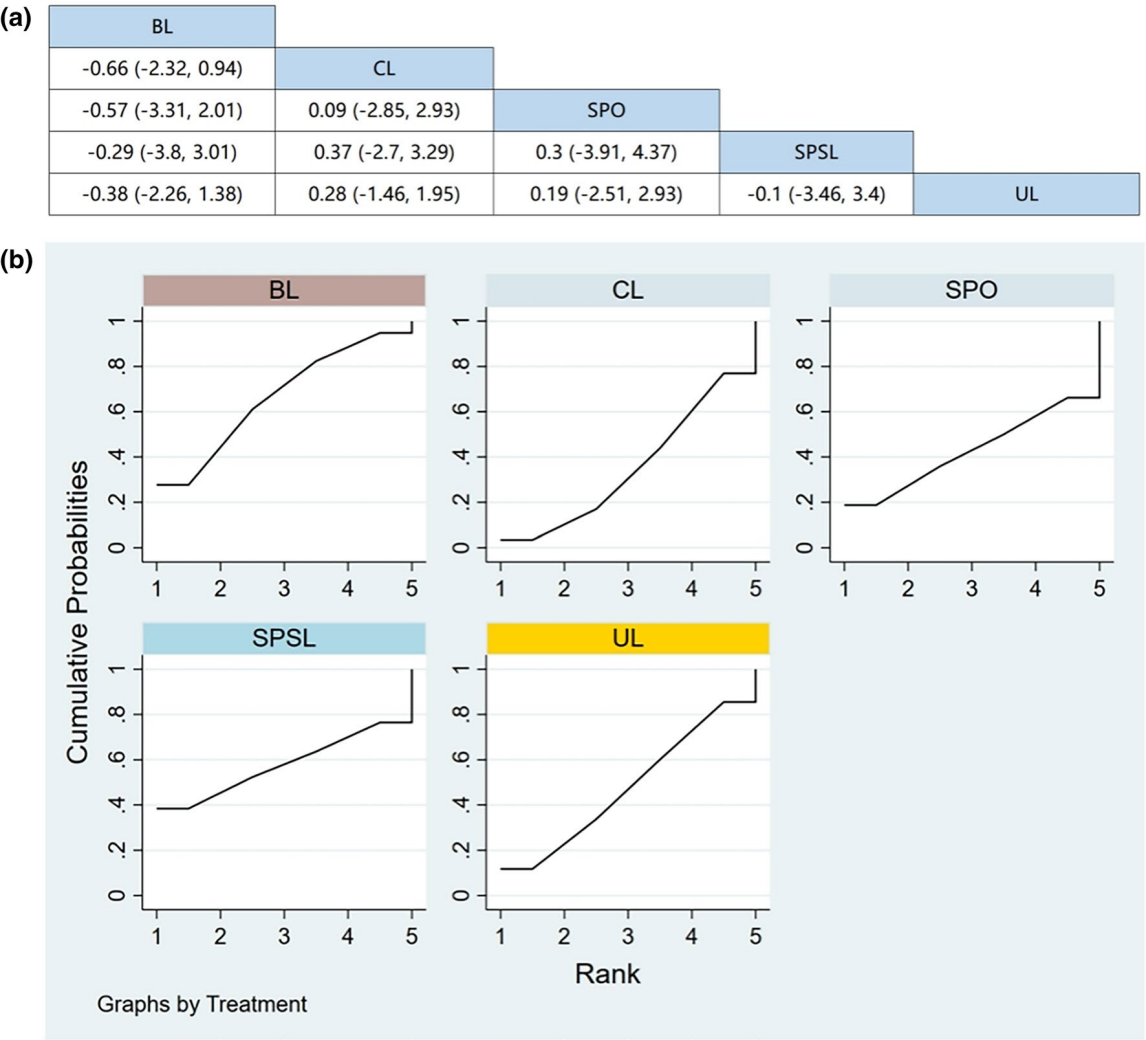
**a** The results of League table for complication rate. **b** Ranking the probability of complication rates

Ten RCTs (including data from 1,022 participants) compared complications among different posterior decompression techniques [30, 32–34, 36, 37, 39, 40, 42, 43]. BL had a lower complication rate compared with other interventions. However, Fig. 10a shows that other than BL, there were no significant differences in complications between any two surgical interventions.

#### Ranking the probability of complication rates

We ranked different posterior decompression techniques using SUCRA. Figure 10b shows the outcome of complication rates, with a higher ranking representing greater safety. The probability of complication rates with various posterior decompression techniques ranked from low to high was as follows: BL (90.3%), SPSL (62.5%), UL (35.9%), CL (50.0%), and SPO (11.3%).

## Discussion

In this systematic review and NMA, we provide a comprehensive overview of the effectiveness and safety of different posterior decompression techniques, including CL, UL, BL, SPSL, and SPO, for LSS. For decreasing back and leg pain, SPO was the most promising surgical option. It was also the best choice for decreasing the ODI score. SSPL had the shortest operation time; however, it was associated with maximum blood loss. SPO and UL were better than any other posterior decompression technique in decreasing blood loss and the length of hospital stay, respectively. Patients who underwent BL had the lowest complication rates after surgery. On combining the effectiveness (VAS and ODI) and safety (blood loss, operation time, and length of hospital stay) of surgery, our research showed that SPO might be the most promising choice of posterior decompression for most patients with LSS. This can likely be attributed to the fact that SPO can maintain spinal stability as much as possible (the lamina is not completely removed and most spinal ligaments are left intact). Meanwhile, this approach also gives a splendid visualization and room to work [46–49]. However, there are few high-quality controlled studies on SPO, which constitute its main limitations. Compared with other posterior decompression techniques, only one RCT has been performed on SPO [34]. Therefore, more high-quality controlled studies with a rigorous design are necessary for SPO.

In recent years, with the rising prevalence of LSS among the middle-aged and older populations, systematic reviews and NMA of related clinical studies have also received increasing attention. For instance, several previous NMA have largely focused on other aspects of the intervention [50–53]. Meanwhile, a few traditional pairwise meta-analyses of posterior decompression techniques have compared BL, UL, and SPSL with CL. Zhang et al. [15]concluded that BL was superior to CL, which was the only significant finding among comparisons of various techniques in their study. Overdevest M et al. [7]did not reach a definite conclusion. Although they are all excellent meta-analyses, they all involved pairwise comparisons of two techniques and did not conclude a comprehensive ranking of posterior decompression techniques. Therefore, we used an NMA to gain a comprehensive ranking of posterior decompression techniques concerning primary and secondary outcomes. We also included a relatively novel posterior decompression technique, SPO. Currently, no NMA is comparing the effectiveness and safety of all current posterior decompression techniques for LSS on a large scale. To the best of our knowledge, this study is the first to use NMA in a large comprehensive statistical analysis to compare different posterior decompression techniques for LSS. Our NMA also has the following strengths: (1) A large number of studies (n = 14) including data from 1,260 patients were included, and all these studies were RCTs. (2) The statistical results had a good consistency. (3) Indirect comparisons among different posterior decompression techniques were used to provide a thorough description of their performance.

Although our NMA included all posterior decompression techniques to gain comprehensive results, there are still several limitations to our study: (1) Some treatments lacked face-to-face comparisons that precluded pairwise analysis and measures of secondary outcomes, such as reoperation rate and instability rate, were incomplete in some cases; and were thus not included.

## Conclusion

This NMA demonstrated that for LSS, SPO was the most promising choice of routine surgery to improve the functional status compared with other posterior decompression techniques. Nevertheless, given that each posterior decompression technique has its upsides and downsides, the surgeon should choose the most appropriate technique considering the patient's situation. In the future, more high-quality studies evaluating the effectiveness and safety of different posterior decompression techniques for patients with LSS are warranted.

### Supplementary Information

Below is the link to the electronic supplementary material.

Supplementary Material 1.

## Data Availability

No datasets were generated or analysed during the current study.
