# Supplementary material for: Comparative effects of different posterior decompression techniques for lumbar spinal stenosis: a systematic review and Bayesian network meta-analysis
Source: J Orthop Surg Res. 2024 Jul 20;19:417. doi: 10.1186/s13018-024-04792-y (PMC11264886; doi:10.1186/s13018-024-04792-y)

**Supplementary Data.**

**Search Strategy;**

**PUBMED （362）**

(((((spinal stenosis[MeSH Terms]) OR (lumbar spinal stenosis[Title/Abstract])) OR (spinal stenosis[Title/Abstract])) OR (neurogenic claudication[Title/Abstract])) AND ((((((((((((((((((((((((Surgical Procedures, Operative[MeSH Terms]) OR (Operative Procedures[Title/Abstract])) OR (Operative Procedure[Title/Abstract])) OR (Procedure, Operative[Title/Abstract])) OR (Procedures, Operative[Title/Abstract])) OR (Surgical Procedure, Operative[Title/Abstract])) OR (Operative Surgical Procedures[Title/Abstract])) OR (Procedure, Operative Surgical[Title/Abstract])) OR (Procedures, Operative Surgical[Title/Abstract])) OR (Surgical Procedures[Title/Abstract])) OR (Procedure, Surgical[Title/Abstract])) OR (Procedures, Surgical[Title/Abstract])) OR (Surgical Procedure[Title/Abstract])) OR (Operative Surgical Procedure[Title/Abstract])) OR (Laminectomy[MeSH Terms])) OR (Laminectomies[Title/Abstract])) OR (Laminotomy[Title/Abstract])) OR (Laminotomies[Title/Abstract])) OR (Osteotomy[MeSH Terms])) ) OR (Minimally Invasive Surgical Procedures[MeSH Terms])) OR (Minimally Invasive[Title/Abstract])) OR (Minimal Surgical Procedure[Title/Abstract])) OR (Minimal Surgical Procedures[Title/Abstract]))) AND ((Randomized Controlled Trial[Publication Type]) OR (Randomized[Title/Abstract]))

**Cochrane Central Register of Controlled Trials (541)**

#1 MeSH descriptor: [Spinal Stenosis] explode all trees

#2 (neurogenic claudication)

#3 (lumbar spinal stenosis)

#4 #1 OR #2 OR #3

#5 MeSH descriptor: [Osteotomy] explode all trees

#6 MeSH descriptor: [Laminectomy] explode all trees

#7 (minimally invasive)

#8 ("endoscopy")

#9 ("decompression")

#10 #5 OR #6 OR #7 OR #8 OR #9

#11 #4 AND #10

**Embase Session Results (626)**

#19 #12 AND #13 AND #17

#18 #12 AND #13 AND #17

#17 #14 OR #15 OR #16

#16 randomized

#15 'randomized controlled trial'

#14 'randomized controlled trial'/exp

#13 #6 AND #12

#12 #7 OR #8 OR #9 OR #10 OR #11

#11 decompression

#10 'endoscopy'

#9 minimally AND invasive

#8 laminectomy

#7 osteotomy

#6 #1 OR #2 OR #3 OR #4 OR #5

#5 'lumbar spinal stenosis'

#4 'neurogenic claudication'

#3 spinal AND stenoses

#2 spinal AND ('stenosis'/exp OR stenosis)

#1 spinal AND stenosis

**Web of science （479）**

1:(((TS=(spinal stenosis)) OR TS=(lumbar spinal stenosis)) OR TS=(canal stenosis)) OR TS=(neurogenic claudication)

2:((((((((((((((((((((((TS=(Surgical Procedures, Operative)) OR TS=(Operative Procedures)) OR TS=(Operative Procedure)) OR TS=(Procedure, Operative)) OR TS=(Procedures, Operative)) OR TS=(Surgical Procedure, Operative)) OR TS=(Operative Surgical Procedures)) OR TS=(Procedure, Operative Surgical)) OR TS=(Procedures, Operative Surgical)) OR TS=(Surgical Procedures)) OR TS=(Procedure, Surgical)) OR TS=(Procedures, Surgical)) OR TS=(Surgical Procedure)) OR TS=(Operative Surgical Procedure)) OR TS=(Laminectomy)) OR TS=(Laminectomies)) OR TS=(Laminotomy)) OR TS=(Laminotomies)) OR TS=(Osteotomy)) OR TS=(minimally invasive)) OR TS=(endoscopy)))

3: (TS=(Randomized Controlled Trial)) OR TS=(Randomized)

4: #1 AND #2 AND #3

|  | The Global inconsistency | ＞0.05 | DIC of Model of consistency | DIC of Model of inconsistency | the difference is less than 5 |
| --- | --- | --- | --- | --- | --- |
| VAS-back pain | 0.4955 | yes | 30.01581 | 30.11745 | yes |
| VAS-leg pain | 0.9633 | yes | 30.40511 | 30.6998 | yes |
| ODI | 0.2143 | yes | 41.63688 | 42.72321 | yes |
| Operation time | 0.4331 | yes | 36.95984 | 36.80795 | yes |
| Duration of hospital stay | 0.5861 | yes | 26.09904 | 26.06517 | yes |
| Blood loss | 0.2875 | yes | 29.75590 | 29.3998 | yes |
| Complication | 0.2592 | yes | 35.05558 | 35.64703 | yes |

**Supplementary Table 1** The results of compareing the deviance information criteria (DIC) between the consistency and inconsistency models

**Supplementary Figure 1.** The results of [forest map](javascript:;) for all outcomes.

a)The results of [forest map](javascript:;) for VAS of the back pain.


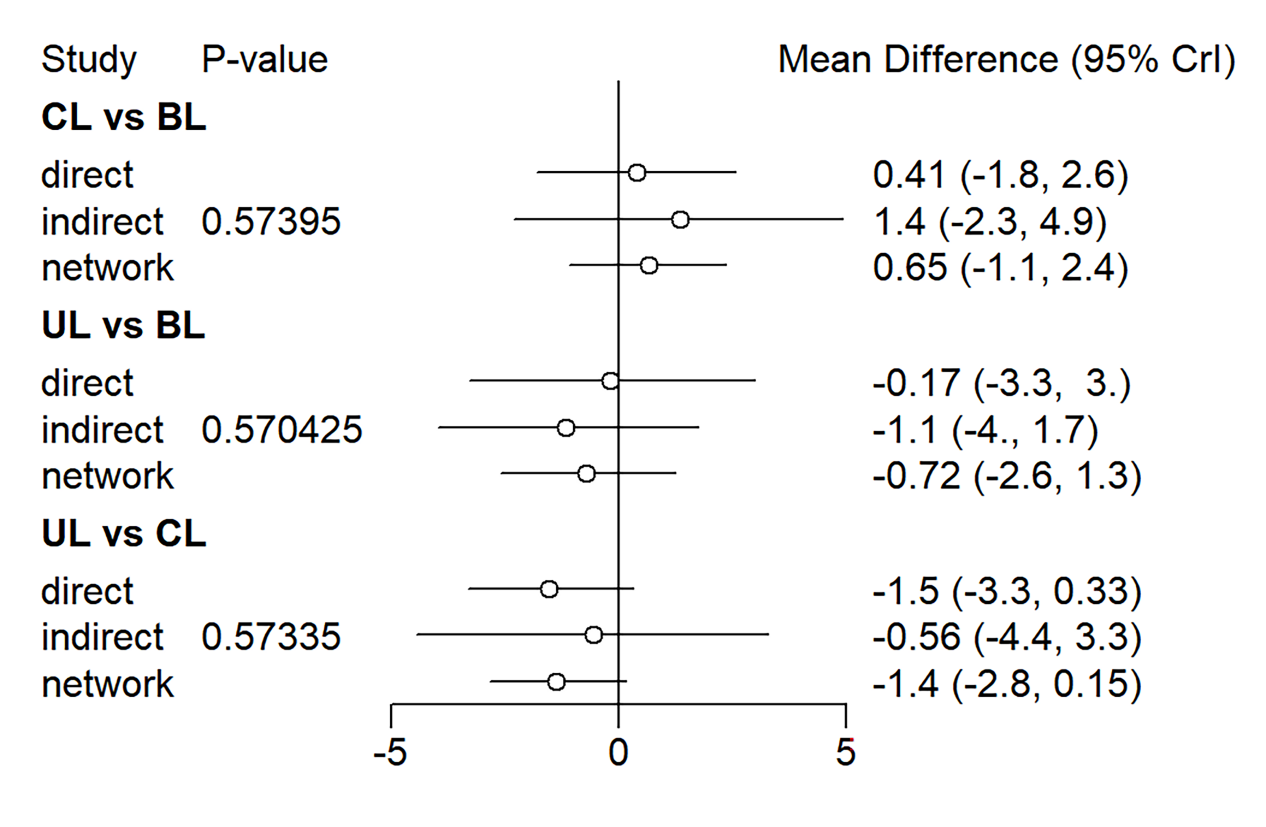


b) The results of [forest map](javascript:;) for VAS for leg pain.


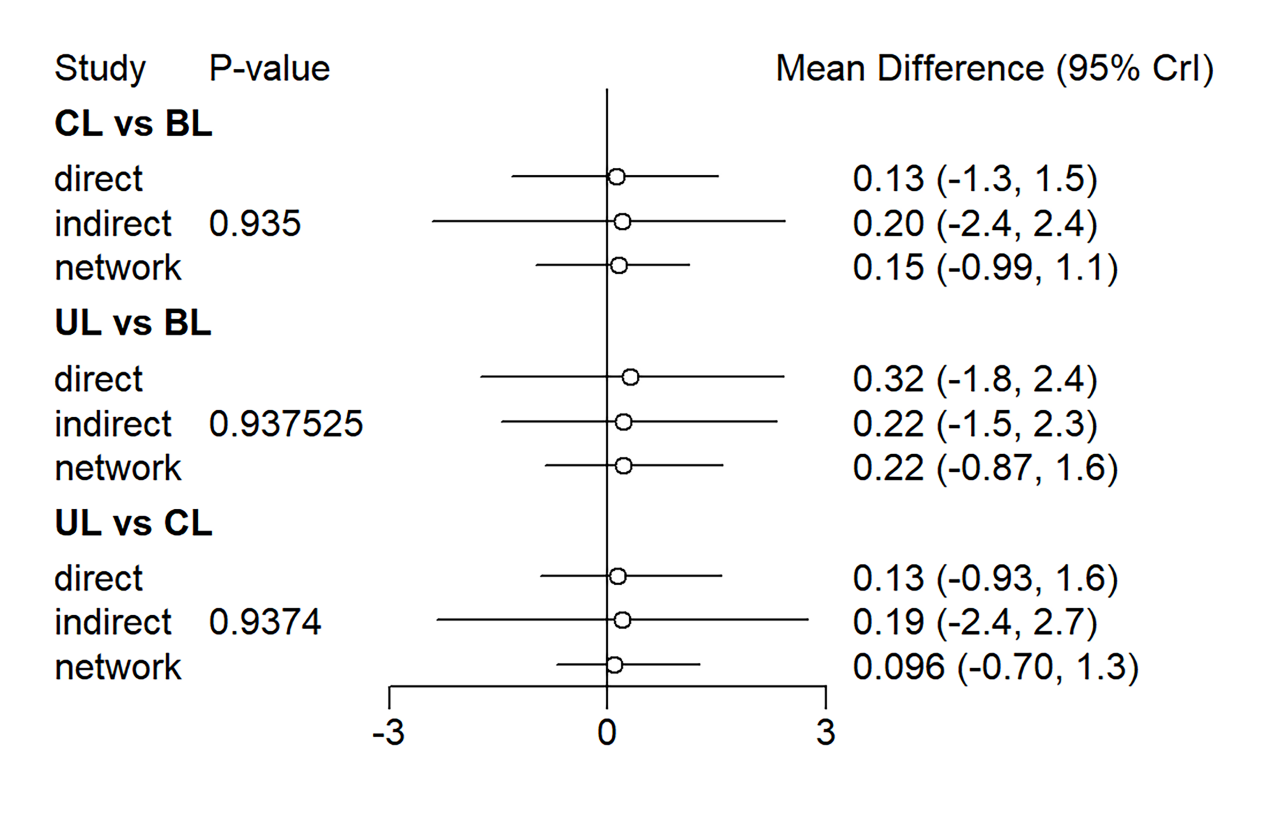


c) The results of [forest map](javascript:;) for ODI.


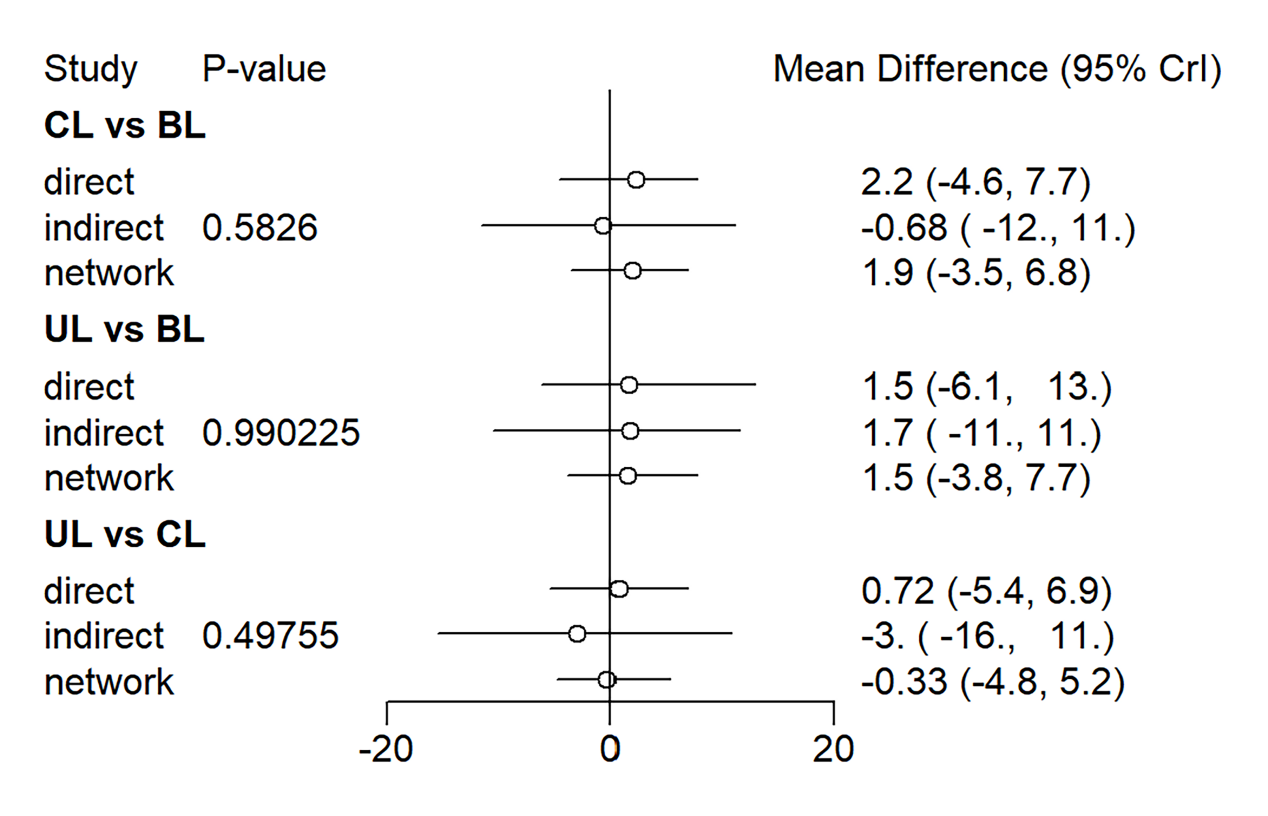


d) The results of [forest map](javascript:;) for blood loss.


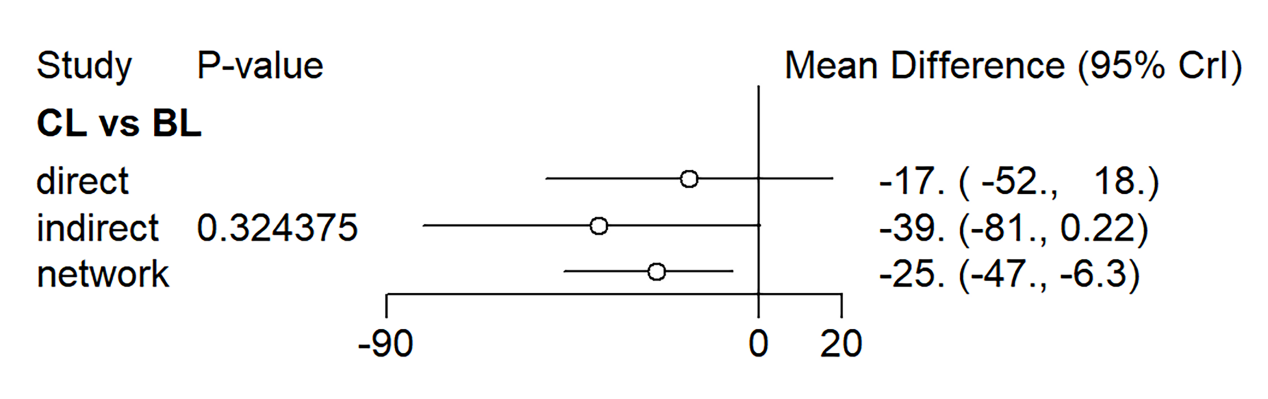


e) The results of [forest map](javascript:;) for operation time.


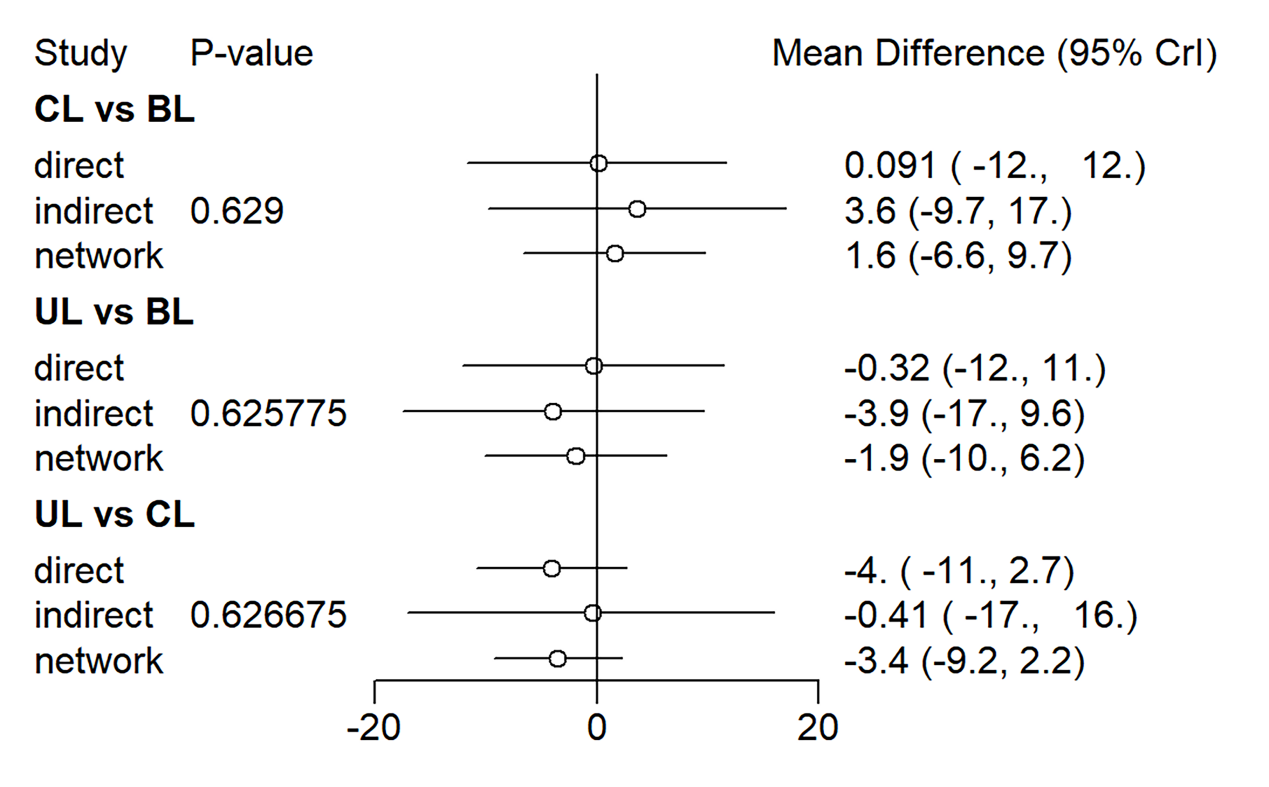


f) The results of [forest map](javascript:;) for hospitalization time.


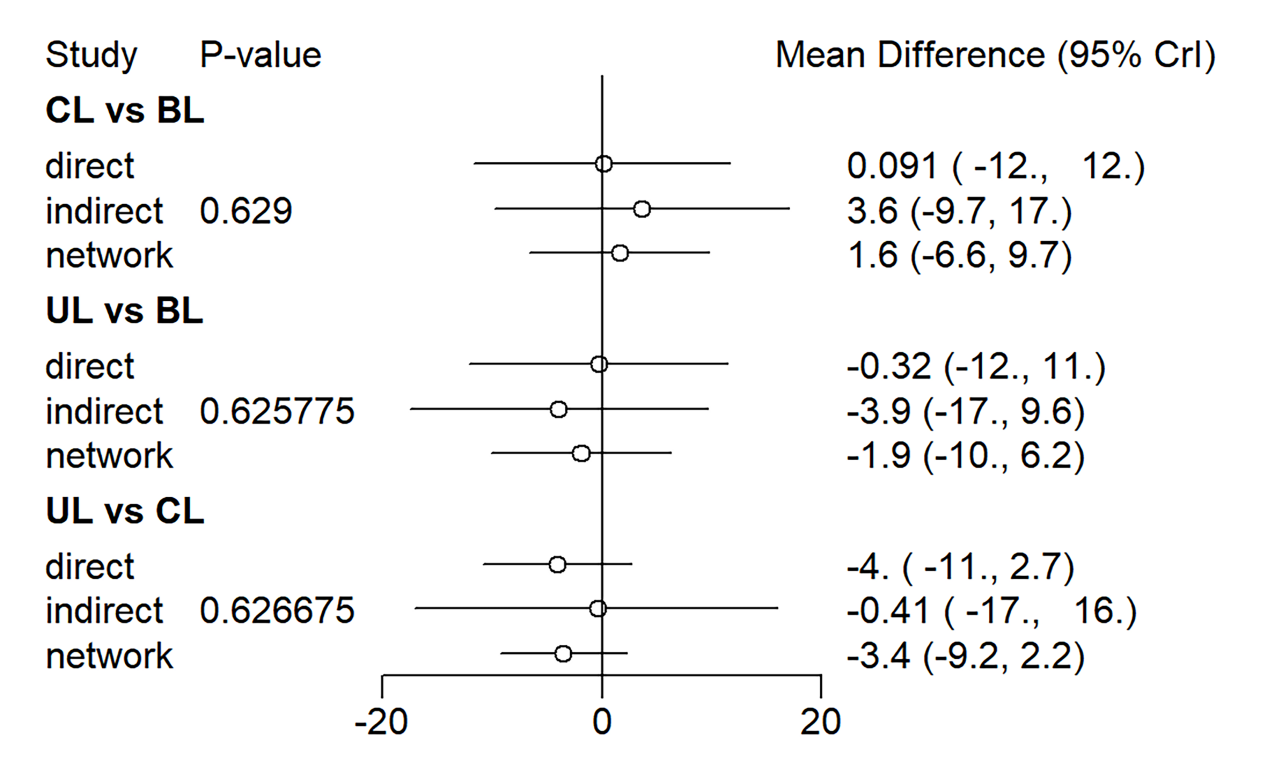


g) The results of [forest map](javascript:;) for complication rates.


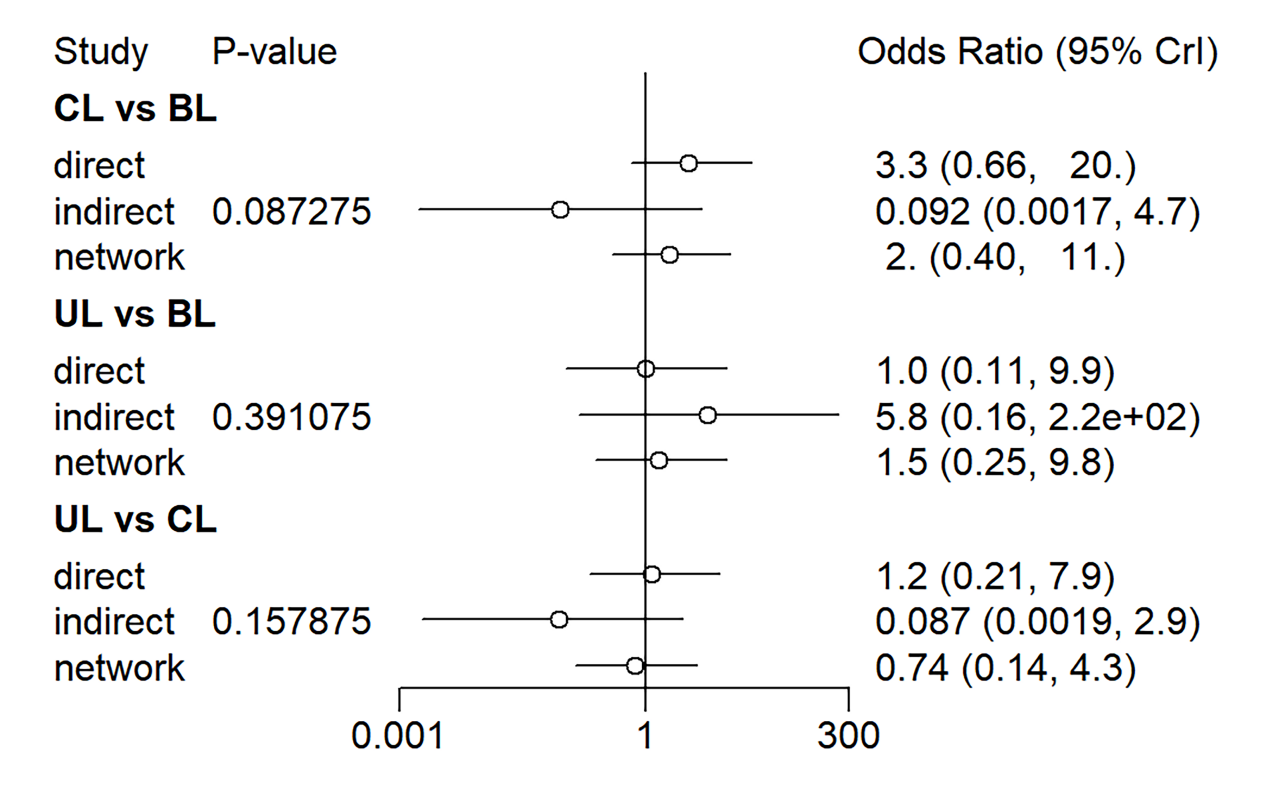


**Supplementary Figure 2.** The network plot of all outcomes.

1. VAS-back pain


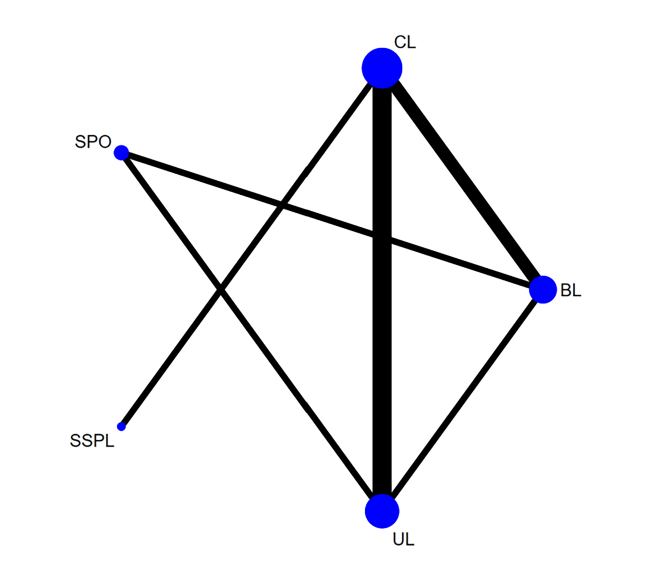


1. VAS-leg pain


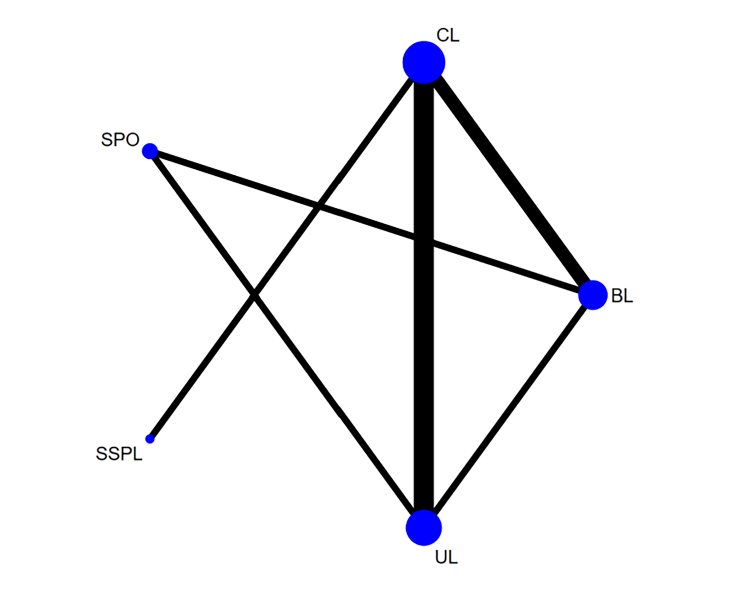


1. ODI


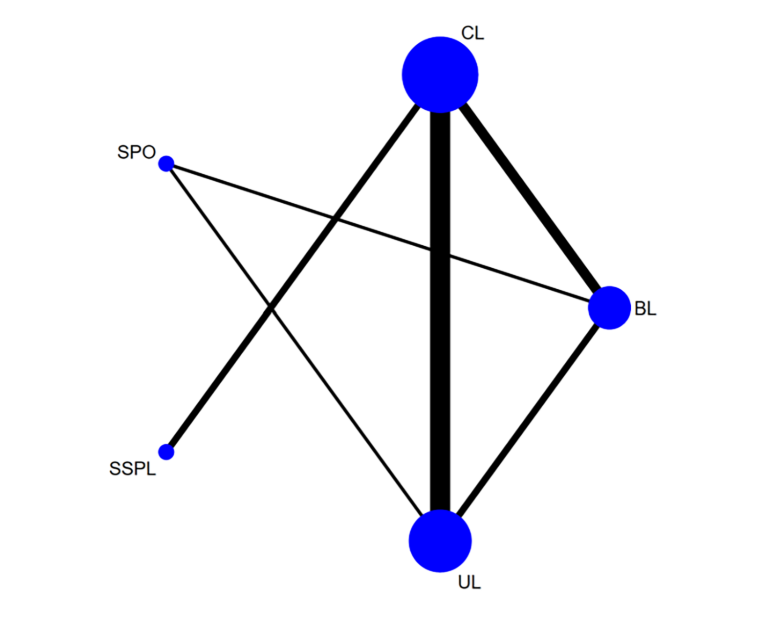


1. Operation time


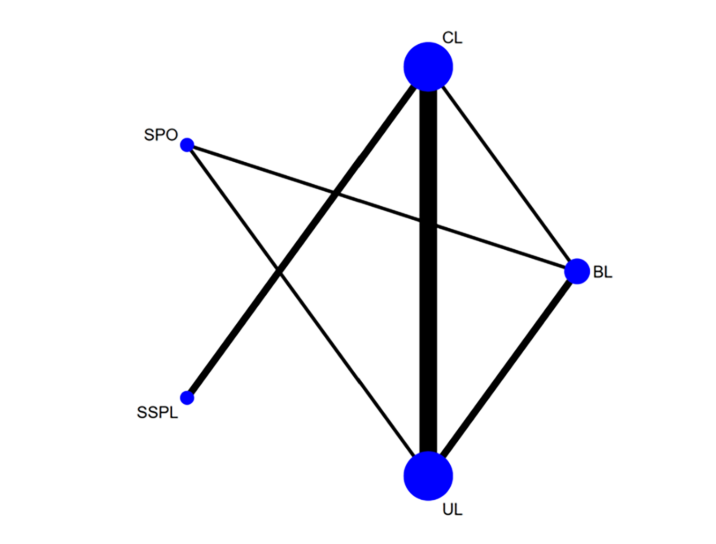


1. Blood loss


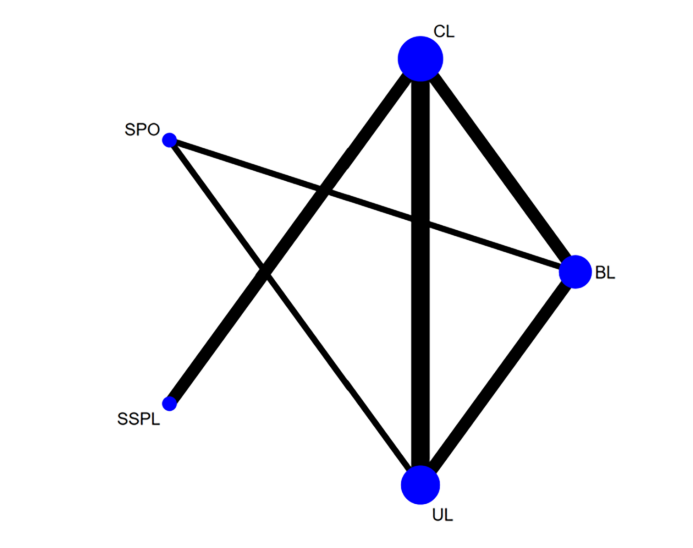


1. During of hospital stay


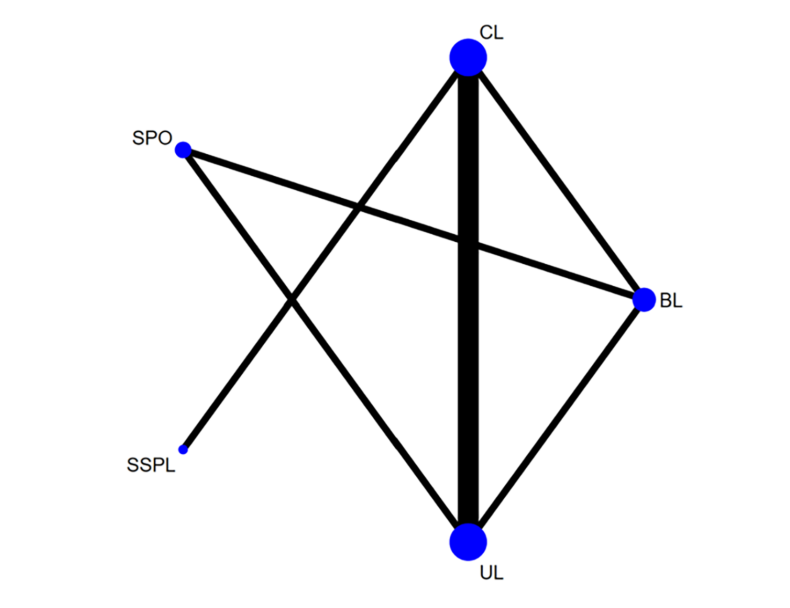


1. Complication


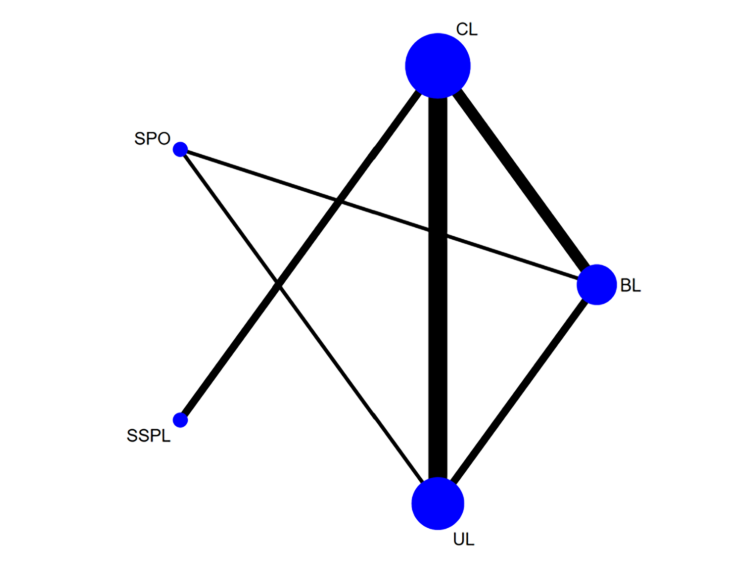


**Supplementary Figure 3.** The funnel plot all outcomes

1. VAS-back pain


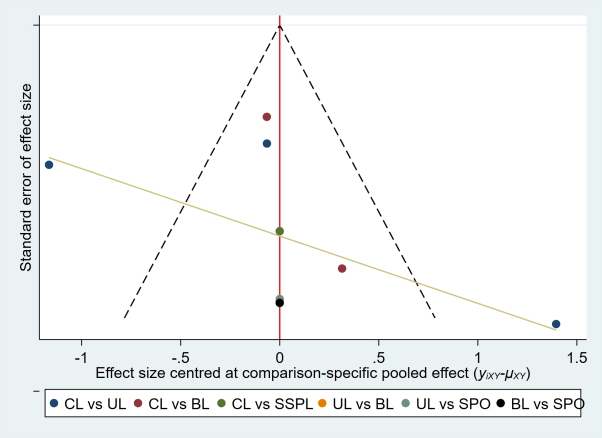


1. VAS-leg pain


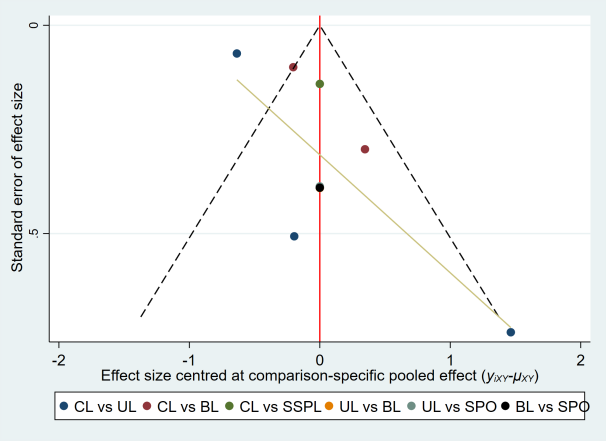


1. ODI


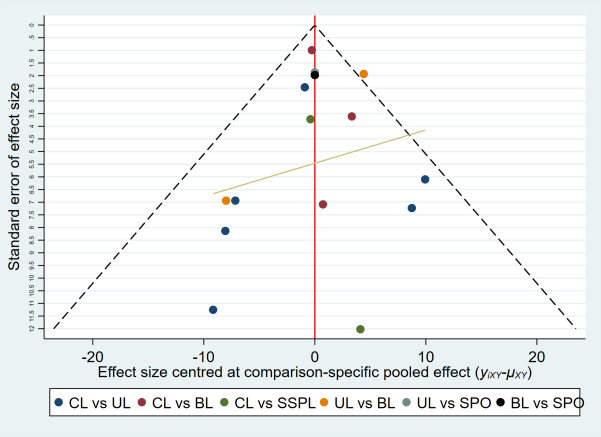


1. Operation time


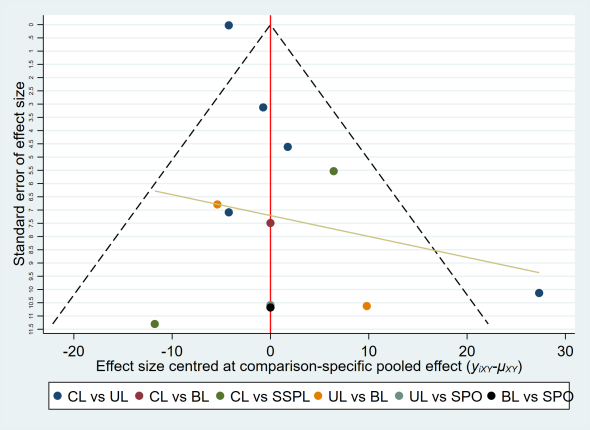


1. Blood loss


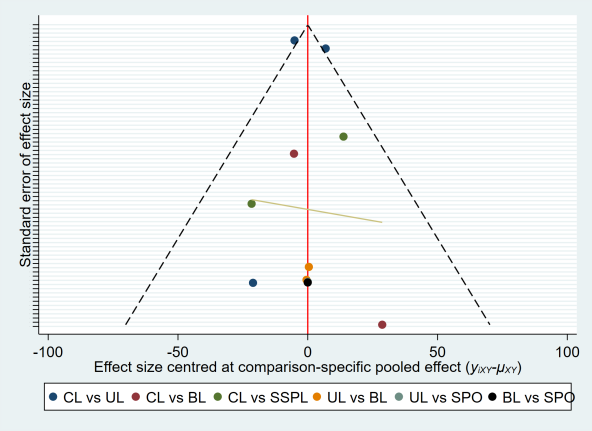


1. During of hospital saty


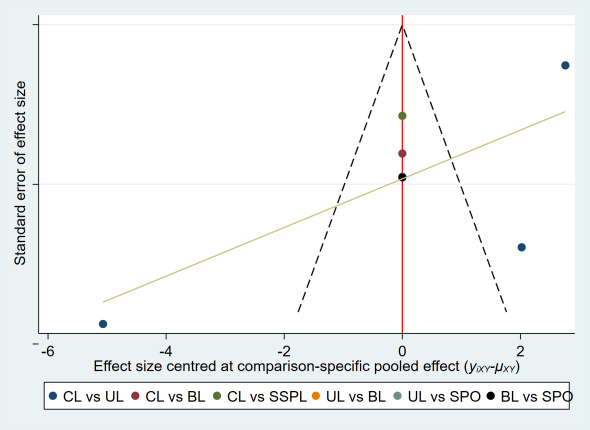


1. Complication


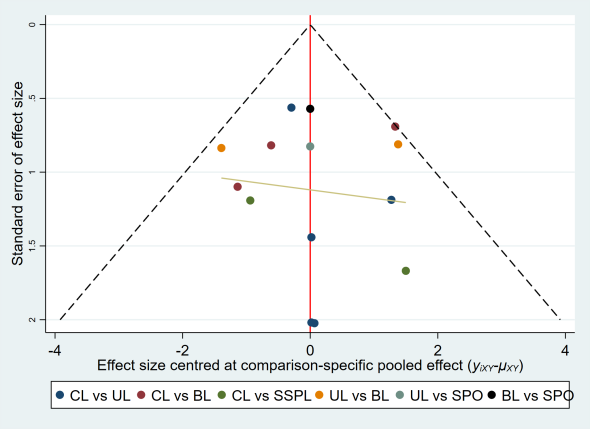

Supplement: Supplementary file 1 — Supplementary Material 1. [file 13018_2024_4792_MOESM1_ESM.docx]
